# Co-Producing Neurodivergence-Informed System Change: Establishing a Novel Trust-Wide Medical Specialist Neurodivergence Advisory Role and Framework in Mental Health Services in Essex

**DOI:** 10.1192/bjo.2026.11367

**Published:** 2026-06-30

**Authors:** Catherine Dakin, Julia Hopper/Caro, Glenn Westropp

**Affiliations:** 1 Essex Partnership University Foundation Trust, Colchester, United Kingdom; 2 Lampard Inquiry, London, United Kingdom

## Abstract

**Aims:**

Autistic and other neurodivergent people experience substantial health inequalities, including poorer mental health outcomes, barriers to accessing care, and premature mortality (autistic people are 7 times more likely to attempt suicide than non-autistic people). These preventable outcomes reflect systemic inequities rather than autism itself. There is, therefore, need for a radical new approach to ward-, service- and organisational-level change to address this profound, unmet need within mental healthcare systems.

Aim: to create a novel Trust-wide Neurodivergence Specialist Advisor role within Essex Partnership University NHS Foundation Trust (EPUT), to improve quality, safety and across all services and age groups through co-produced partnership working to drive professional development, system transformation and an essential cultural shift around all neurodivergence.

**Methods:**

This service-development initiative involved partnership working between an Autistic/ADHD psychiatrist and a lived-experience expert, grounded in participatory and co-production principles. Activity domains will include: bespoke patient and service clinical consultations; supporting Patient Safety Incident Investigations, Inquest, LeDeR panels, and Prevention of Future Death processes to turn learning into transformational change;delivery of co-produced training addressing workforce knowledge gaps (e.g. National Autism Trainer Programme – additional and complementary to Oliver McGowan Mandatory Training); service development aligned with organisational governance quality priorities; and collaborative partnership working with local authority and system partners to address interface gaps where neurodivergent people are excluded from support and care pathways.

**Results:**

Significant drive leading to the successful implementation of this new frameworkand advisory role is an outcome of sustained lived-experience advocacy and campaigning, reflecting the influence, expertise, and leadership of the co-authoring lived-experience partner, reinforcing the value and principles of participatory and co-production in healthcare design.Experiential knowledge is essential to shaping equitable systems, not as an adjunct perspective.

Structured evaluation is planned at six months, including audit of activity and service impact (including patient safety data), thematic analyses, and review through executive governance processes as defined by EPUT’s “Working with Neurodivergence” group. This will explore perceived accessibility, workforce ability and confidence, system responsiveness, and identification of previously unrecognised gaps across service pathways. Findings will inform iterative refinement of the role and contribute to organisational learning regarding scalable neurodivergence-informed service transformation.

**Conclusion:**

Embedding neurodivergence expertise and lived experience at organisational level represents a scalable approach to addressing structural inequities in mental healthcare for neurodivergent people. This model emphasises transformational co-production, workforce development, and cross-system collaboration as mechanisms. Early implementation insights will guide wider adoption and contribute to improving outcomes for neurodivergent populations.

